# When Heat Is on: Posttranslational Regulation of Flowering Under Warming Climates—Its Significance and Potential Coping Strategies

**DOI:** 10.3390/biology15130988

**Published:** 2026-06-23

**Authors:** Zeeshan Nasim, Nouroz Karim

**Affiliations:** Department of Life Sciences, Korea University, Seoul 02841, Republic of Korea; nourozkarim@korea.ac.kr

**Keywords:** global warming, flowering, climate change, posttranslational regulation, LLPS

## Abstract

**Simple Summary:**

Rising global temperatures accelerate plant flowering, threatening agricultural productivity. While the transcriptional regulation of flowering is well understood, protein-level mechanisms such as protein degradation, ubiquitination, and liquid–liquid phase separation are now recognized as equally important mechanisms enabling rapid and reversible temperature responses. This review focuses on recently identified protein-level thermosensory modules that are important for flowering responses to elevated temperatures. Insights from these mechanisms open new avenues for engineering climate-resilient crops by targeting protein stability and phase separation behavior. However, most of the work in this field is from the model plant *Arabidopsis*, and the conservation of these posttranslational mechanisms has yet to be tested in crops.

**Abstract:**

Global warming poses serious threats to plant reproduction and agricultural productivity by affecting the timing of flowering, a critical developmental transition. Although transcriptional regulation of flowering pathways has been extensively studied, posttranslational and protein-level regulatory mechanisms are gaining increasing attention as important thermosensory switches enabling rapid and reversible responses to temperature fluctuations. These mechanisms include temperature-dependent protein degradation, ubiquitination, liquid–liquid phase separation of intrinsically disordered proteins, protein sequestration, and dynamic protein–protein interactions. This review summarizes current understanding of posttranslational flowering time regulation under high-temperature conditions, focusing on the major interconnected thermosensory modules, such as the temperature-dependent proteostasis of floral repressors and the emergence of temperature-responsive liquid–liquid phase separation (LLPS) of intrinsically disordered proteins (IDPs). Recent discoveries indicate that temperature-responsive flowering relies not only on transcriptional networks but also on dynamic protein-level regulatory mechanisms, including ubiquitination, proteasomal degradation, and liquid–liquid phase separation. However, the fact that these mechanisms have not been validated in crop species leaves their translational potential an open question.

## 1. Introduction

Flowering is a critical developmental transition in the plant life cycle that determines reproductive success, seed production, and yield. Given its importance, the timing of this transition must be tightly regulated and synchronized with environmental conditions to maximize reproductive fitness. Several environmental factors can influence flowering; among them, temperature has emerged as a critical regulator of flowering time [1,2], especially under the current global warming conditions. Average global surface temperature has already increased by approximately 1.1 °C above pre-industrial levels, with an increasing frequency and intensity of heat waves [3]. Elevated temperatures impair reproductive development, shorten the grain-filling period, and reduce yield across major crops. In wheat, heat stress during anthesis and grain filling accelerates phenological development, shortens the grain-filling period, and reduces yield by 3–8% per °C above optimal temperatures [4]. In rice, high night-time temperatures during grain filling produce chalky grains with lower quality and yield losses of up to 7.1–8.1% per °C [5,6]. Temperatures above 35 °C reduce pollen germination and tube growth in maize, leading to kernel abortion and yield losses [7,8]. In soybean, heat stress during the reproductive phase causes yield losses of up to 25% [9]. In tomato, temperatures above 32 °C during anthesis negatively affect pollen tube growth and sugar accumulation, reducing both yield and quality [10].

The pronounced effects of elevated temperatures on reproductive development and yield underscore the need to understand the molecular mechanisms governing flowering, under changing environmental conditions. Plants have evolved complex regulatory networks that modulate flowering in response to both exogenous and endogenous cues. To date, over 400 genes have been functionally characterized as flowering regulators and categorized into eight genetically distinct pathways, including autonomous, vernalization, photoperiod, and ambient-temperature pathways [11]. Most of these pathways converge on a set of common genes, known as the floral integrators, such as the floral promoters *FLOWERING LOCUS T* (*FT*), *TWIN SISTER OF FT* (*TSF*), and *SUPPRESSOR OF OVEREXPRESSION OF CONSTANS 1* (*SOC1*), as well as their upstream repressors *FLOWERING LOCUS C* (*FLC*), *FLOWERING LOCUS M* (*FLM*), and *SHORT VEGETATIVE PHASE* (*SVP*) [11,12]. However, transcriptional regulation alone does not fully explain the rapid, reversible, and quantitatively precise nature of temperature-responsive flowering.

FLC, FLM, and SVP are major floral repressors that interact to form floral repressor complexes, which fine-tune flowering across a broad range of temperatures [2,13,14]. *FLC* encodes an MADS-box transcription factor that acts as a potent floral repressor in many plant species, including *Arabidopsis* [15,16]. *FLC* is epigenetically regulated by Polycomb Repressive Complex 2 (PRC2) in response to prolonged cold exposure (vernalization) and transcriptionally regulated by the autonomous pathway [17,18,19]. FLC represses flowering by directly repressing the floral promoter genes *FT* and *SOC1* [20]. FLM acts as a temperature-responsive floral repressor that is primarily regulated at the posttranscriptional level via temperature-sensitive alternative pre-mRNA splicing [13,21]. *FLM* predominantly produces the functionally repressive *FLM-β* splice variant at lower temperatures, while increasing temperatures shift splicing toward the generation of *FLM-δ* isoform, thereby relieving floral repression [13,22,23]. SVP is another highly potent floral repressor in the ambient-temperature pathway and plays a critical role in regulating temperature-responsive flowering, as *svp* mutants show temperature-insensitive early flowering across a broad range of temperatures (5–27 °C) [13]. Unlike FLC and FLM, SVP is primarily regulated at the posttranslational level. Its transcript levels are largely unperturbed across temperatures, whereas SVP protein abundance rapidly declines at higher temperatures through 26S proteasome-dependent degradation [13,24]. These three major floral repressors interact with each other to form floral complexes that directly repress the downstream floral promoter genes *FT* and *SOC1* [14,21,25].

Alongside these MADS-box TFs, Phytochrome B (phyB) and EARLY FLOWERING 3 (ELF3) are also important floral repressors that modulate flowering in response to light and temperature [2]. PHYB is a prominent red light photoreceptor that indirectly represses flowering under different photoperiodic conditions, primarily by destabilizing the floral activator CO during the morning [26,27]. In contrast, ELF3 is a nuclear scaffold protein and a core component of the evening complex (EC), alongside ELF4 and LUX ARRHYTHMO (LUX), which repress downstream floral promoters such as *GIGANTEA* (*GI*) and *FT* [28,29]. PHYB and ELF3 directly interact, and this interaction enhances ELF3 protein stability by preventing its COP1-mediated proteasomal degradation [28,30]. phyB acts as a thermostat in *Arabidopsis* [31,32]. PhyB is converted from the inactive Pr state to the active Pfr state by both light and temperature; red light drives Pr-to-Pfr photoactivation, whereas the active Pfr form can spontaneously revert to the inactive Pr state through a high-temperature-accelerated process known as thermal reversion [33].

Liquid–liquid phase separation (LLPS) has recently emerged as a functionally important regulatory hub for fine-tuning protein activity. For instance, LLPS provides a specialized microenvironment that enables EMBRYO DEFECTIVE 1579 (EMB1579) to carry out diverse biological functions, including the regulation of flowering time through *FLC* [34] and the control of cell division required for root meristem maintenance through interactions with root developmental regulators [35]. PhyB has been shown to undergo phase separation to assemble liquid-like droplets, and the phase behavior of these droplets is directly regulated by temperature through its N-terminal extension (NTE) region [36]. High temperatures abolish phyB droplet formation and hence renders phyB nonfunctional at elevated temperatures [31], thereby promoting early flowering.

Although transcriptional mechanisms have long dominated studies of temperature-responsive flowering, recent discoveries reveal that protein-level regulation provides equally important thermosensory control. These mechanisms enable rapid, reversible, and highly dynamic responses to fluctuating temperatures. Given that core floral transition networks are highly conserved across angiosperms—particularly central thermal and photoperiodic regulators such as SVP, FLM, phyB, CO, and the evening complex component ELF3—the underlying posttranslational regulatory mechanisms are likely to be broadly conserved as well [37,38]. Therefore, mechanistic insights gained from model species may provide a valuable framework for identifying and engineering analogous thermosensory pathways in crop plants to enhance resilience under future climate-warming scenarios. The following sections discuss the major protein-level thermosensory modules that coordinate flowering under elevated temperatures.

## 2. The SVP–FLM Thermoregulatory Repressor Module

### 2.1. SVP as a Central Floral Repressor of Temperature-Responsive Flowering

SVP is one of the most important repressors of temperature-responsive flowering [39], especially under cooler ambient temperatures. SVP binds directly to the promoter regions of floral integrator genes *FT* and *SOC1,* causing their repression and thereby suppressing the reproductive transition [13,24]. A key breakthrough in ambient-temperature flowering research was the discovery that SVP protein levels strongly depend on temperature [13]. SVP accumulates at low temperatures but is rapidly degraded at higher temperatures, while its transcript levels remain largely unchanged [13], suggesting that warm-temperature-mediated regulation occurs primarily at the posttranslational level.

High temperatures accelerate SVP protein turnover via the 26S proteasome pathway. The half-life of the SVP protein significantly decreases as temperature increases, establishing a direct connection between temperature perception and flowering repression. The biological significance of this mechanism is demonstrated by *svp* mutants, which exhibit temperature-insensitive flowering across a broad range of temperatures [13]. This allows SVP to function as a core temperature-responsive protein switch, enabling flexible developmental responses.

### 2.2. Temperature-Dependent FLM Isoforms and Protein Interaction Dynamics

FLM, a major floral repressor, acts as a primary interaction partner of SVP. *FLM* undergoes temperature-dependent alternative splicing to generate two major isoforms, *FLM-β* and *FLM-δ* [23]. At cooler temperatures, plants accumulate higher levels of FLM-β, which forms a stable nuclear repressor complex with SVP [21]. This SVP–FLM-β complex strongly represses *FT* and *SOC1* transcription. At higher temperatures, plants accumulate increased levels of the FLM-δ splice variant. FLM-δ lacks floral repressor activity and alters SVP stability and localization. FLM-δ promotes the cytoplasmic retention and ubiquitination of SVP, accelerating SVP degradation [21]. In short, warm temperatures regulate SVP-mediated flowering through two posttranslational mechanisms: reduced SVP protein stability and temperature-dependent alterations in SVP interaction partners [21]. Together, these posttranslational regulatory mechanisms confer high thermosensitivity to flowering regulation in *Arabidopsis*.

### 2.3. SVP Degradation via the CRL3-LFH1-UBC15 Complex

**Figure 1:**
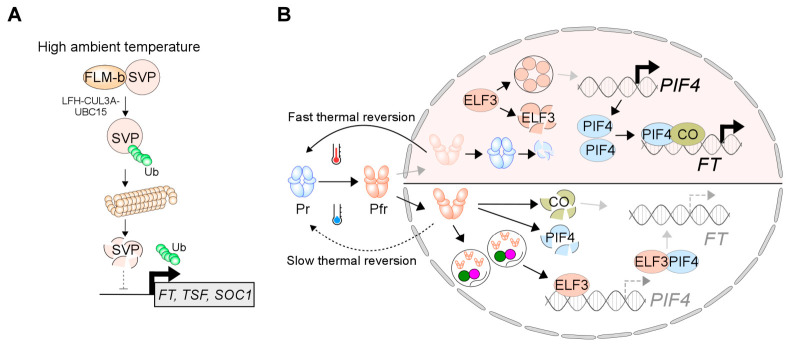
Temperature-dependent posttranslational regulation of flowering. (**A**) High temperature destabilizes the floral repressor SVP through LFH1–CUL3A–UBC15-mediated ubiquitination and 26S proteasomal degradation, relieving repression of *FT*, *TSF*, and *SOC1*. (**B**) Elevated temperature accelerates phyB reversion from the active Pfr to the inactive Pr state, affecting downstream regulators ELF3, PIF4, and CO. At low-temperature conditions (lower right), Pfr-phyB promotes degradation of CO and PIF4 and stabilizes ELF3 in nuclear photobodies, suppressing flowering. At warm temperature (upper right), Pfr-to-Pr reversion prevents photobody formation, and phyB is degraded, stabilizing CO and PIF4 to induce *FT* expression and promote flowering. Black arrows indicate induction, whereas gray arrows indicate failure to induce.

SVP has been shown to be regulated by the 26S proteasomal system in a temperature-dependent manner. However, the E3 ubiquitin ligase responsible for this process was identified only recently by Jin and colleagues [40]. Through protein pull-down screens of SVP interactors at different temperatures, they identified a BTB/POZ-domain protein, named LATE FLOWERING AT HIGH TEMPERATURE 1 (LFH1) as a high-temperature-specific interactor of SVP. LFH1 functions as a substrate adaptor within a CULLIN3–RING E3 ligase (CRL3) complex, bridging SVP to CUL3A and the cognate E2 ubiquitin-conjugating enzyme UBC15 [40] (Figure 1A).

The *lfh1* and *cul3a* mutants phenocopied SVP overexpression lines, as they displayed late flowering only at high ambient temperatures, highlighting that the regulatory complex is specifically required for high temperature-accelerated flowering. Furthermore, the SVP protein was highly stable in *lfh1* mutants, without any difference in transcript levels. Interestingly, LFH1 protein accumulates to higher levels at higher temperatures. Because LFH1 promotes SVP degradation, this creates a feed-forward loop: rising temperatures increase LFH1 levels, which clear more SVP, further promoting flowering in a self-amplifying manner. This self-reinforcing mechanism distinguishes the CRL3–LFH1 module from simpler degradation pathways and likely contributes to the robustness and irreversibility of high-temperature-induced flowering. However, the role of FLM-β in modulating CRL3-LFH1-UBC15-mediated SVP degradation remains unclear and requires further research. While SVP degradation represents a major thermoregulatory mechanism, temperature-responsive flowering is also controlled through the stabilization and activity of transcriptional activators such as PIF4.

## 3. PIFs and Temperature-Regulated Protein Stability

PIF4 is a well-studied central integrator of temperature signaling, thermomorphogenesis, and flowering regulation [41]. At higher temperatures, PIF4 protein accumulates to higher levels and activates genes associated with hypocotyl elongation and flowering induction [42,43]. Among the flowering genes, PIF4 directly activates *FT* expression at high temperatures, especially under short-day photoperiods [42]. PIF4 activity is largely regulated at the protein level through dynamic interactions with photoreceptors such as phyB [44] and ubiquitin ligases such as BOPs [45].

The phyB-PIF4 module serves as a key hub for integrating light and temperature signals, with protein-level regulation of PIF4 protein being critical for thermomorphogenesis and flowering modulation. PIF4 acts as a key growth-promoting transcription factor that is stabilized at higher ambient temperatures. At elevated temperatures, phyB is less active and therefore impaired in its ability to interact with PIF4 and sequester it in the cytoplasm. This temperature-dependent reduction in phyB functionality allows PIF4 to accumulate in the nucleus and induce its target genes, thereby promoting flowering and hypocotyl elongation [31,32]. In addition to its nuclear translocation, PIF4 protein stability is modulated by the ubiquitin–proteasome system, with several E3 ubiquitin ligases, including BLADE-ON-PETIOLE (BOP) 1 and 2, triggering PIF4 degradation in a temperature-dependent manner. BOP proteins function as substrate adaptors in CUL3-based E3 ubiquitin ligase complexes that ubiquitinate PIF4 and hence promote its degradation via the 26S proteasome pathway. The efficiency of this degradation is fine-tuned by temperature and light conditions [45]. Under cool-to-moderate ambient temperatures (<23 °C), the BOP-mediated 26S proteasomal system rapidly degrades PIF4 to keep cell elongation in check. In contrast, elevated temperatures dampen this BOP-mediated degradation [45]. However, the fact that PIF4 ubiquitination and degradation are not completely abolished in *bop* mutants suggests the involvement of additional E3 ligases or proteolytic pathways that modulate PIF4 stability [45]. Contrary to BOPs, a recent report identified LIGHT-RESPONSE BRIC-A-BRACK/TRAMTRACK/BROAD (LRB) E3 ubiquitin ligases as positive regulators of PIF4 accumulation [46]. LRB1 and LRB2 physically interact with PIF4 and stabilize it, likely by inhibiting its degradation by phyB and possibly by counteracting the action of unknown E3 ubiquitin ligases. In *lrb123* mutants, PIF4 protein levels were significantly reduced under both optimal and warm temperatures, confirming that LRBs are essential for normal PIF4 stability.

Additionally, LRBs also negatively regulate ELONGATED HYPOCOTYL 5 (HY5) by promoting its degradation via the 26S proteasomal pathway. HY5 functionally antagonizes PIF4 by competing for binding to the promoters of shared target genes [47]. LRB-mediated degradation of HY5 relieves this inhibitory effect on PIF4 resulting in enhanced activation of PIF4 target genes. Thus, LRBs constitute a dual regulatory model that simultaneously stabilize PIF4 and promotes the degradation of its functional antagonist, HY5, resulting in the fine-tuning of PIF4 activity [46]. However, additional regulators of PIF4 stability are likely to exist. Furthermore, as PIFs have functional redundancies, they are likely to undergo similar protein-level regulation, although this has yet to be experimentally validated. Collectively, these findings establish ubiquitin-mediated protein turnover as a key regulatory mechanism underlying temperature-responsive flowering, a theme that is conserved across multiple E3 ligase complexes in plants. Among these, the CONSTITUTIVE PHOTOMORPHOGENIC 1 (COP1) pathway represents one of the best-characterized mechanisms linking protein degradation to temperature-responsive flowering.

## 4. COP1-Mediated Protein Degradation at High Temperature

The COP1–SUPPRESSOR OF PHYA (SPA) E3 ubiquitin ligase complex integrates ambient-temperature signals from the photoperiodic, circadian, and hormonal flowering pathways into the plant flowering modules. Elevated temperatures promote the nuclear accumulation and importin-α/β-mediated transport of COP1, leading to the formation of tetrameric COP1–SPA holocomplexes [48]. This regulatory module is activated in the nucleus during the daytime and modulates proteasomal degradation cascades that target distinct master regulators of the photoperiodic, circadian, and hormonal flowering pathways.

In photoperiodic and circadian pathways, COP1 recognizes its target proteins through its WD40-repeat domains, and this interaction results in the polyubiquitination of HY5 and GI, marking them for rapid degradation via the 26S proteasome system [49,50]. The accelerated turnover of GI prevents it from forming a complex with FLAVIN-BINDING KELCH REPEAT F-BOX 1 (FKF1), which normally promotes the degradation of the flowering repressor CYCLING DOF FACTOR 1 (CDF1) [51]. Consequently, the accumulation of CDF1 transcriptionally represses *FT*, thereby delaying flowering under extreme heat [52]. Concurrently, the COP1–SPA1 module directly interacts with DELLA repressors, such as REPRESSOR OF GA1 (RGA) and GA-INSENSITIVE (GAI), leading to their degradation in *Arabidopsis thaliana*, as well as that of MdRGL1a in *Malus domestica*, under high-temperature stress independently of the canonical GA pathway [53,54]. Interestingly, this posttranslational regulation has divergent, species-specific developmental effects, accelerating flowering in some while causing severe floral repression in other species [53,54]. In addition to protein degradation, plants exploit the biophysical properties of proteins to sense temperature through phase separation.

## 5. Phase Separation: A New Paradigm for Temperature Sensing

Cells contain several membrane-less compartments and biomolecular condensates that offer unique microenvironments for macromolecules to concentrate and play their specific biological roles [55]. LLPS is a process in which a solution separates into two or more distinct liquid phases that coexist, thereby forming biomolecular condensates [56]. Phase separation primarily occurs for proteins with multivalency conferred by intrinsically disordered regions (IDRs) and/or multiple folded domains [57]. IDPs lack stable tertiary structures under physiological conditions and have emerged as a versatile platform for sensing and responding to environmental and developmental cues. Unlike properly structured proteins with fixed conformational states, IDPs are structurally flexible and undergo reversible, stimulus-responsive phase transitions, subsequently dissolving when favorable conditions are restored.

In recent years, LLPS has gained considerable attention as an increasing number of studies have demonstrated its biological significance in modulating plant development and responses to environmental cues [58]. For instance, the major floral repressor FLC has been shown to be regulated by LLPS. The transcriptional repressor REDUCED VERNALIZATION RESPONSE 1 (VRN1) undergoes LLPS driven by its two B3 domains and an IDR, resulting in dynamic nuclear condensates that results in the epigenetic silencing of *FLC* [59]. Similarly, the RNA-binding protein FLOWERING CONTROL LOCUS A (FCA) contains a prion-like domain (PrLD) that assembles into nuclear condensates with the help of coiled-coil protein FLL2, which acts as a scaffold. These condensates selectively recruit RNA 3′-end-processing factors that polyadenylate *COOLAIR* antisense transcripts, epigenetically repressing *FLC* and inducing flowering [60]. In contrast, the IDR-containing transcriptional activator SUF4 acts as a posttranslational temperature sensor and undergoes temperature-dependent phase separation, forming nuclear condensates that stabilize *FLC* transcription at warm temperatures to repress flowering. At cooler temperatures, these condensates dissolve, lowering the barrier to vernalization [61]. However, this review focuses on the role of LLPS in regulating temperature-responsive flowering at higher temperatures.

### 5.1. GI LLPS Modulate SVP-Mediated Temperature-Responsive Flowering

Recent studies have shown how plants utilize LLPS to sense and integrate temperature cues into endogenous photoperiodic pathway [62]. The FLAVIN-BINDING, KELCH REPEAT, F-BOX 1 (FKF1) protein and its interacting partner GI, core components of daytime photoperiodic signaling, play important roles in overriding photoperiodic constraints to drastically accelerate flowering. Under long-day (LD) conditions, the FKF1 and GI protein levels both peak in the late afternoon, resulting in the formation of FKF1–GI complexes that promote the degradation of CDF proteins and hence prevent CDF-mediated repression of *CO* and *FT*, thereby modulating day length-dependent flowering [51]. FKF1–GI complexes also physically interacts with CO protein, stabilizing it and enhancing its ability to induce *FT* under LD conditions [51,62].

This FKF1–GI complex also plays an important role in accelerating flowering time at elevated temperatures under non-inductive photoperiodic conditions by inducing *FT* transcript levels [63]. This high-temperature-induced *FT* expression is governed by the LLPS of GI protein. Lower ambient temperatures promote GI phase separation, resulting in its sequestration and functional inactivation within nuclear condensates. At warm ambient temperatures, FKF1 accumulates to high levels and interacts with GI, reversing GI phase separation and bringing GI back to its active state. The FKF1-GI complex then physically interacts with SVP, recruiting it to an E3 ubiquitin ligase complex for ubiquitination and subsequent 26S proteasomal degradation [63]. The rapid degradation of SVP relieves its direct transcriptional repression of *FT* and thereby facilitates flowering under warm conditions. Although this work clearly demonstrates that FKF1-mediated reversal of GI phase separation controls the turnover of a major floral repressor, several questions remain, particularly regarding the relative contributions of CO and PIF proteins to this high-temperature-mediated *FT* induction and floral acceleration.

### 5.2. The ELF3 Prion-like Domain as a Thermosensor: Phase Separation Links Temperature to Flowering Time Control

ELF3, a core scaffold component of the evening complex, contains a polyglutamine (polyQ) repeat embedded within its prion domain (PrD) [64,65]. The length of this polyQ repeat-containing PrD region positively correlates with high temperature-induced hypocotyl elongation and early flowering. Variations in the length of PrD result in differential temperature responsiveness and hence adaptation to climate conditions with different ambient temperatures. ELF3 orthologs from species naturally adapted to warmer climates, such as *Brachypodium distachyon*, lack a PrD and are therefore unable to confer temperature-responsive flowering when expressed in *Arabidopsis*. Swapping the *Arabidopsis* PrD with the *B. distachyon* PrD abolished the thermosensitivity of chimeric *Arabidopsis* ELF3 (AtELF3-BdPrD), suggesting that the PrD itself is the functional thermosensory unit. This PrD was shown to be important for regulation of flowering at elevated temperatures. Overexpression of *Arabidopsis* ELF3 did not alter flowering, implying that increased ELF3 protein levels alone are not sufficient to repress flowering [66]. The ELF4, another EV complex protein and binding partner of ELF3, stabilizes ELF3 activity. Under low-temperature conditions, ELF4 becomes dispensable, whereas overexpression of ELF4 maintains ELF3 in an active state and enables its chromatin-binding ability even at a warmer temperature of 27 °C [66].

### 5.3. PhyB Photobodies Integrate Light and Temperature Signals Through Phase Separation

Phytochrome B (phyB) acts as a dual receptor for both light and temperature cues in plants. Chen and colleagues [36] showed that light-activated phyB undergoes LLPS to form nuclear photobodies (PBs). The capacity for LLPS is modulated by two structurally distinct domains with complementary roles: the C-terminal domain, which mediates light-independent self-oligomerization and hence establishes the multivalency required for condensate assembly, and the intrinsically disordered N-terminal extension (NTE), which functions in phase separation and enables light-dependent regulation of condensate formation. Light-induced conformational changes expose the buried NTE and thereby enable NTE-mediated multivalent interactions that promote oligomerization and subsequent PB assembly. Deletion of the NTE abolishes both droplet formation and phyB activity, while replacing it with heterologous IDRs restores its condensate formation but eliminates light-dependent regulation, suggesting that NTE possesses both a general LLPS capacity and a sequence-specific function in connecting condensation to light status.

High-temperature-mediated regulation of phyB activity occurs through a mechanistically distinct pathway that converges on the same LLPS platform. phyB condensates are intrinsically thermosensitive; they disassemble as temperature rises and reassemble reversibly upon cooling, indicating that temperature directly modulates condensate dynamics independent of Pfr-to-Pr dark reversion. This thermosensitivity is entirely dependent on the NTE region, as loss of the NTE makes the condensates temperature-insensitive, which cannot be rescued by heterologous IDRs, suggesting that the NTE contains sequence-specific thermosensory capacity. Once formed, phyB photobodies selectively recruit interacting transcription factors, including PIF3 and PIF4, into concentrated microreactors, and transgenic plants expressing NTE-deleted phyB lose elevated temperature-induced hypocotyl elongation despite retaining partial light responses (Figure 1B). Collectively, phyB photobodies act as flexible sensory condensates that decode two distinct environmental cues through separate mechanisms. Light is processed through allosteric conformational changes that gate condensate assembly, whereas temperature is sensed through NTE-dependent regulation of condensate stability, enabling a single receptor to precisely integrate both signals [36]. While phyB photobodies sense temperature through condensate dynamics, plants have evolved parallel chromatin-based mechanisms that translate thermal signals into changes in gene expression. One of the most prominent chromatin-based thermosensory mechanisms involves the histone variant H2A.Z.

## 6. H2A.Z-Mediated Chromatin Remodeling in Temperature-Responsive Flowering

The histone variant H2A.Z is a key mediator of this chromatin-based thermosensory response, marking temperature-responsive genes across the genome [67]. In *Arabidopsis thaliana*, H2A.Z is encoded by *HISTONE H2A* (*HTA*) *8*, *HTA9*, and *HTA11* [68]. Its deposition into target chromatin is mediated by the ATP-dependent SWR1 chromatin-remodeling complex (SWR1-C), which catalyzes the exchange of H2A–H2B dimers for H2A.Z–H2B dimers [69,70]. H2A.Z incorporation occurs through an ATP-dependent process involving nucleosome priming, DNA unwrapping, and the subsequent replacement of H2A-containing dimers [71]. In *Arabidopsis*, SWR1-C consists of several conserved subunits, including PHOTOPERIOD INDEPENDENT EARLY FLOWERING 1 (PIE1), ACTIN-RELATED PROTEIN 6 (ARP6), SWR COMPLEX 6 (SWC6), ARP4, YEAST AF9 (YAF9), and other conserved subunits [69,72,73,74,75]. Recruitment of SWR1-C to target loci occurs through several mechanisms, including the recognition of AT-rich DNA elements by SWC4 and recruitment by ALFIN-LIKE proteins through H3K4me3 recognition [76,77]. Emerging evidence further suggests that LLPS-mediated transcriptional condensates may facilitate local concentration of chromatin remodelers at warm-temperature-responsive loci [78]. However, this hypothesis has yet to be experimentally validated.

Genetic and genomic studies have established H2A.Z as a central regulator of temperature-responsive transcription. A forward genetic screen identified *arp6* mutants that exhibited aberrant *HSP70* expression at low temperatures, demonstrating that H2A.Z normally represses heat-responsive genes under non-inductive conditions [68]. Consistently, genome-wide chromatin immunoprecipitation (ChIP) assays revealed the rapid depletion of H2A.Z from the gene body and transcription start site regions of warm-temperature-responsive genes in response to elevated temperatures [79]. This process occurs within 15 min of transfer to 27 °C and requires HEAT SHOCK FACTOR 1 (HSF1), which promotes H2A.Z-to-H2A exchange at heat-responsive loci [79]. The antagonistic relationship between H2A.Z and HSF1 provides an effective mechanism to prevent inappropriate transcription at low temperatures while enabling rapid activation upon warming [79].

Beyond warm-temperature-responsive gene expression, H2A.Z plays important roles in flowering time regulation. Mutants lacking H2A.Z or SWR1-C components, including *pie1*, *arp6*, *swc6*, *arp4*, and *yaf9*, exhibit early flowering and reduced sensitivity to ambient-temperature changes [69,72,75,80,81,82], phenocopying plants grown under warm conditions. These findings suggest that H2A.Z eviction is an early thermosensory event that promotes the floral transition.

Mechanistically, H2A.Z regulates the expression of several flowering repressors, including *FLC*, *MAF4*, and *MAF5* [80,81,82,83]. However, the early flowering phenotypes of H2A.Z-deficient plants cannot be fully explained by these targets alone. Warm-temperature-induced eviction of H2A.Z from the promoter region of *FT* likely facilitates access by transcriptional activators such as CO and PIF4 [42,84,85], thereby enhancing *FT* expression and promoting flowering.

Despite significant progress, important questions remain unanswered. The molecular mechanisms governing the fate of evicted H2A.Z–H2B dimers, their redistribution between chaperone-bound pools and chromatin, and the spatiotemporal dynamics of H2A.Z turnover remain poorly understood. Furthermore, whether LLPS-mediated condensates regulate H2A.Z deposition or eviction at thermoresponsive loci has not been experimentally tested [78].

## 7. Future Directions: Engineering Resilience Through Posttranslational Precision

### 7.1. Manipulation of SVP-FLM Interaction

Biasing *FLM* splicing toward FLM-β by modifying splicing elements could reduce SVP cytoplasmic retention and subsequently its proteolytic turnover, preventing global warming-induced acceleration of flowering. The K53 and K165 residues are critical ubiquitination sites on SVP. They can be precisely altered through base editing to produce conservative lysine-to-arginine substitutions, which could stabilize SVP at elevated temperatures and hence mitigate heat-induced flowering acceleration in crops where extending the vegetative growth period under warming is agronomically valuable, such as leafy vegetables, forage grasses, or biomass crops. In addition, producing dominant-negative LFH1 variants or interrupting the LFH1–SVP interaction by manipulating the Kh2C domain of SVP or/and the C-terminal region of LFH1 could stabilize SVP in crops where premature flowering under warming reduces yield. However, because SVP is a major developmental regulator, its stabilization may impose trade-offs between prolonged vegetative growth and reproductive fitness. Excessive SVP activity could delay floral transition and reduce reproductive performance; therefore, engineering SVP stability would require careful optimization to balance heat resilience with agronomic productivity.

### 7.2. Manipulating the LLPS of ELF3 and PhyB

LLPS is a highly promising and rapidly evolving aspect underlying plants responses to diverse endogenous and environmental factors, including high temperatures. A promising avenue for climate-resilient agriculture is the manipulation of phase separation properties to engineer crops with tailored temperature sensitivities.

Variations in the length of the PrD region result in differential temperature responsiveness and, subsequently, adaptation to climates with different ambient temperatures. Because ELF3 functions as a direct thermosensor within the EV complex through a polyglutamine (polyQ) repeat embedded in its PrD, precision genome-editing strategies could be used to modulate the temperature threshold of its phase separation. Using tools like CRISPR-Cas9 to precisely shorten the polyQ or introduce stabilizing amino acid substitutions within the PrD could prevent the heat-induced LLPS and its subsequent transcriptional deactivation, which triggers premature flowering. By structurally hardening ELF3 against thermal inactivation, EV complex-mediated repression can be maintained at warmer temperatures, delaying premature reproductive transition. This provides a potential framework for engineering temperature-responsive traits in crops, although its agronomic benefits remain to be experimentally validated.

PhyB functions as a thermosensor and undergoes light-activated LLPS to form subnuclear photobodies. To suppress the selective recruitment of downstream transcription factors like PIF4, future bioengineering strategies should focus on precision editing of this regulatory module. CRISPR-Cas9 tools could be used to alter specific residues within the regulatory NTE, thereby structurally stabilizing phyB photobodies against heat-induced dissolution, maintaining active transcriptional regulation and preventing the premature, heat-accelerated flowering that typically penalizes crop yields. Furthermore, mapping the natural variation in phyB LLPS dynamics across diverse plant species will supply a valuable genetic toolkit for marker-assisted selection, establishing a tunable framework to safeguard global food security in a warming climate. A recent study has reported phyB variants, phyB^G564E^ and phyB^G515E^, that stabilize the active Pfr form and were able to form high-temperature-insensitive nuclear photobodies, hence preventing high-temperature-induced thermal reversion and subsequent heat-induced hypocotyl elongation in *Arabidopsis* and stem elongation in rice [86]. Although the effects of these variants on flowering outcome have yet to be tested, they are likely to resist warm-temperature-induced flowering acceleration as well, offering a potential strategy for developing climate-resilient crops.

Despite recent progress in understanding temperature-responsive flowering at the posttranslational level, the picture remains incomplete. Several critical questions remain unresolved, and targeted work on posttranslational regulation under elevated temperatures is still needed. Notably, most posttranslational mechanisms identified to date in response to elevated temperatures promote flowering. To our knowledge, feedback loops that reset thermosensory posttranslational switches have not yet been identified. Future studies should investigate whether plants possess posttranslational mechanisms that actively prevent precocious flowering under warming conditions. Furthermore, most posttranslational research has been carried out in model plants, primarily *Arabidopsis thaliana*, and whether these regulatory mechanisms are conserved in agriculturally important crop species remains largely untested.

### 7.3. Beyond Arabidopsis: Posttranslational Regulation of Flowering in Crops Remains Largely Uncharted

Most of our understanding of posttranslational regulation of flowering has been derived from work in *Arabidopsis*, leaving a significant gap in knowledge for crop species. While orthologs of key regulators such as PIFs, FLC, SVP, FT, and CO exist in other plant species, whether the same ubiquitin-mediated degradation, phase separation, or temperature-sensitive protein interaction mechanisms operate in these species remains largely untested. Closing this gap will require systematic validation across diverse species and is critical for translating these mechanisms into crop-improvement strategies.

Recent evidence suggests that LLPS may represent a broadly conserved regulatory principle across plant species [87]. Large-scale proteomic screens have identified hundreds of potential phase separation proteins across diverse plant species, including rice, maize, wheat, and tomato. Interestingly, while the primary sequences of IDRs show limited conservation, their presence is highly conserved, indicating that the capacity for LLPS may be under positive selection throughout plant evolution [87]. However, these experiments were performed using a cell-free system and hence require validation under in vivo conditions.

Functional evidence linking LLPS to flowering regulation in crops has begun to emerge. In rice, the prion-like protein (PrLP) EHD6 (EARLY HEADING DATE 6) recruits YTH07, an m^6^A reader protein, to form ribonucleoprotein (RNP) condensates. These granules facilitate the sequestration of the mRNA of the flowering repressor *OsCOL4* (*CONSTANS-like 4*), which is critical for reducing *OsCOL4* abundance and accelerating flowering [88]. Nevertheless, mechanistic insights into how elevated temperatures influence condensate dynamics and flowering-related posttranslational networks in crops remain scarce.

## 8. Conclusions

Collectively, recent advances demonstrate that temperature-responsive flowering is governed not only by transcriptional regulation but also by an interconnected network of posttranslational mechanisms. Protein degradation, ubiquitination, sequestration, and phase separation collectively enable plants to rapidly adjust flowering time in response to fluctuating temperatures. Understanding and harnessing these processes may facilitate the development of climate-resilient crops adapted to a warming climate. However, despite substantial progress, our understanding of these mechanisms remains incomplete. Most protein-level thermosensory pathways discussed in this review have been characterized primarily in *Arabidopsis thaliana*, and their conservation, biological significance, and agronomic utility in crop species remain largely unexplored. Furthermore, the mechanistic integration of ubiquitin-mediated proteolysis, phase separation, chromatin remodeling, and temperature-sensitive protein interactions within a unified thermosensory network is not yet fully understood. Addressing these knowledge gaps will be essential for translating fundamental discoveries into practical crop-improvement strategies under future climate scenarios.

## Data Availability

No new data were created or analyzed in this study. Data sharing is not applicable to this article.
